# Inactivation Efficacy of 405 nm LED Against *Cronobacter sakazakii* Biofilm

**DOI:** 10.3389/fmicb.2020.610077

**Published:** 2020-11-27

**Authors:** Yixiao Huang, Quanwei Pei, Ruisha Deng, Xiaoying Zheng, Jialu Guo, Du Guo, Yanpeng Yang, Sen Liang, Chao Shi

**Affiliations:** ^1^College of Food Science and Engineering, Northwest A&F University, Yangling, China; ^2^Beijing Advanced Innovation Center for Food Nutrition and Human Health, Beijing Technology and Business University, Beijing, China

**Keywords:** *Cronobacter sakazakii*, LED, biofilm, ATR-FTIR, disinfectant

## Abstract

The objectives of this study were to evaluate the inactivation efficacy of a 405-nm light-emitting diode (LED) against *Cronobacter sakazakii* biofilm formed on stainless steel and to determine the sensitivity change of illuminated biofilm to food industrial disinfectants. The results showed that LED illumination significantly reduced the population of viable biofilm cells, showing reduction of 2.0 log (25°C), 2.5 log (10°C), and 2.0 log (4°C) between the non-illuminated and LED-illuminated groups at 4 h. Images of confocal laser scanning microscopy and scanning electron microscopy revealed the architectural damage to the biofilm caused by LED illumination, which involved destruction of the stereoscopic conformation of the biofilm. Moreover, the loss of biofilm components (mainly polysaccharide and protein) was revealed by attenuated total reflection Fourier-transformed infrared spectroscopy, and the downregulation of genes involved in *C. sakazakii* biofilm formation was confirmed by real time quantitative PCR analysis, with greatest difference observed in *fliD*. In addition, the sensitivity of illuminated-biofilm cells to disinfectant treatment was found to significantly increased, showing the greatest sensitivity change with 1.5 log reduction between non-LED and LED treatment biofilms in the CHX-treated group. These results indicated that 405 nm LED illumination was effective at inactivating *C. sakazakii* biofilm adhering to stainless steel. Therefore, the present study suggests the potential of 405 nm LED technology in controlling *C. sakazakii* biofilms in food processing and storage, minimizing the risk of contamination.

## Introduction

*Cronobacter sakazakii* is a rod-shaped, non-spore-forming, peritrichous, facultative anaerobic Gram-negative foodborne pathogen, belonging to the *Enterobacteriaceae* family (Stephan et al., 2014). *C. sakazakii* is occasionally detected in meat, vegetables, cereals and dairy (Beuchat et al., 2009). In particular, powdered infant formula has been linked with many *C. sakazakii* infections, according to an epidemiological report (Yan et al., 2012). *C. sakazakii* contamination more frequently causes infection in infants or adults with lowered immunity, suffering from bacteremia, meningitis, necrotizing enterocolitis or other diseases, and a lethality rate of more than 50% has been reported (Ye et al., 2014). Furthermore, surviving patients may suffer further acute neurological complications such as quadriplegia, brain abscesses, neural-development delay, and hydrocephalus.

Biofilms are defined as matrix-enclosed bacterial populations that are adherent to each other and to interfaces or surfaces, including adherent populations within the pore spaces of porous media, as well as microbial aggregates and floccules (Park et al., 2012). Prior studies have shown that biofilms have complex structures that are surrounded by extracellular polymeric substances (EPS), which enhance the resistance of pathogens to external stress (Ling et al., 2020) and even conventional antibiotics (Davies, 2003). *C. sakazakii* can form biofilms on multiple contact surfaces such as stainless steel, glass, and Teflon (Reich et al., 2010). Furthermore, *C. sakazakii* biofilm formed on the food contact surface can provide a physical defense to protect them from stronger external stress such as high voltage and many antibiotics (Kuo et al., 2013). *C. sakazakii* biofilms have become the main source of contamination for food, either directly or indirectly, and therefore pose a serious threat to consumers' health (Bayoumi et al., 2012).

Conventional antibiofilm methods mainly include disinfectants, exogenous detergents (da Silva and De Martinis, 2013), near-infrared radiant heating and ultraviolet (UV) light (Ha and Kang, 2014). The anti-biofilm effect of natural antibiotics has also been reported (Tong et al., 2018). However, the disadvantages of disinfectants and exogenous detergents cannot be ignored since they can induce resistance in pathogens to conventional treatments, which increases the difficulty of eliminating biofilm (Simões et al., 2010). Infrared radiant heating and UV light irradiation may cause the loss of sensitive nutrients from the food and have limitations because of the high capital costs for installation (Harouna et al., 2015). Natural antibiotics are also limited in their use because of their complex extraction process and high cost (Moloney, 2016). Therefore, new methods for eliminating biofilm that are safe, residue-free and efficient, and could be applied to a variety of processing environments, are needed.

A light-emitting diode (LED) is a semiconductor device that can emit monochromatic light in a narrow range of the spectrum. It has many merits such as low-energy, high stability, long service life, availability in various shapes, and small volume (Quishida et al., 2016). LEDs have advanced in response to the growing need for non-thermal, physical methods for food processing, and storage (Krames and Grandjean, 2017). Some molecules, known as photosensitizers, in bacteria will absorb light when exposed to an LED and produce reactive oxygen species (ROS) that in turn react with DNA, proteins, lipids, and other components to produce cytotoxic effects (Luksiene, 2010). In the field of food safety, LEDs were first studied *in vitro* for their effects against several planktonic foodborne pathogens, including *Escherichia coli, Salmonella, Listeria*, and *Staphylococcus aureus* (Ghate et al., 2013; Kumar et al., 2016). In recent years, LED light within the range of 400–520 nm has been demonstrated to have inactivation efficacy against *Streptococcus mutans, L. monocytogenes*, and *Pseudomonas aeruginosa* on the surface of food and packaging materials (McKenzie et al., 2013; Ghate et al., 2019). However, there have been no studies about the inactivation efficacy of LEDs against *C. sakazakii* biofilms or how they exert photodynamic inactivation.

The current study was carried out to evaluate the inactivation efficacy of a 405 nm LED against *C. sakazakii* biofilm. After LED treatment, the population of viable cells in the biofilm was determined and structural damage to the biofilm was examined using confocal laser scanning microscopy (CLSM) and scanning electron microscopy (SEM). Then, attenuated total reflection Fourier-transformed infrared spectroscopy (ATR-FTIR) combined with real time quantitative PCR (RT-qPCR) analysis were performed to further confirm the 405 nm LED inactivation of *C. sakazakii* biofilm. In addition, the change in sensitivity of LED-illuminated biofilm cells to disinfectants such as chlorhexidine (CHX) and cetylpyridinium chloride (CTPC) was determined. A stainless steel sheet was chosen as the contact surface since it is a common material in food processing, storage and consumption. Three common storage and processing temperatures (25, 10, and 4°C) were selected as illumination temperatures. The inactivation efficacy of 405 nm LED illumination against biofilms was evaluated at room temperature (25°C) to simulate commonly encountered conditions. Cooler temperatures (4 and 10°C) were used to simulate retail storage and processing conditions, respectively.

## Materials and Methods

### Creation of a Biofilm Removal Device With a 405 nm LED

The LED illumination system was prepared according to the previous method reported by Ghate et al. (2013). LED intensity was 26 ± 2 mW/cm^2^, as measured by a 405 nm radiometer (UHC405, UVATA Ltd., Hong Kong). An acrylonitrile butadiene styrene (ABS) housing was used to surround the LED system, preventing incoming light. LEDs were attached by a heat sink and cooling fan to avoid overheating. To avoid over-currents, the circuit was connected to a 5 Ω resistor. The distance between the stainless steel fixer and the LED light was set to 4.5 cm to ensure that the entire sheets could be illuminated. To measure changes in the sheet surface temperature during LED illumination in real time, a thermocouple sensor was used.

### Cleaning of the 304 Stainless Steel Sheets

Stainless steel sheets (grade 304, size: 50 × 20 mm, No. 4 light cleaning face) were used in this study as the biofilm attachment surface for *C. sakazakii*. The procedure for cleaning the stainless steel sheets was conducted according to the method described previously (Yang et al., 2015). Briefly, the sheets were immersed in a test tube with ~20 mL of detergent solution and sonicated for 30 min. Then, the sheets were rinsed with sterile deionized water several times to dissipate any remaining material, and were transferred into 20 mL of 70% (v/v) ethanol and sonicated for another 15 min (Kim et al., 2019). After cleaning, the sheets were air-dried and sterilized in a high temperature autoclave for 15 min before use.

### Strain Cultivation and Bacterial Suspension Preparation

*C. sakazakii* strain ATCC 29004 was purchased from the American Type Culture Collection (ATCC, Manassas, VA, USA) and was preserved in tryptone soya broth (TSB, Land Bridge Technology, Beijing, China) containing 20% (v/v) glycerol at −80°C. For activation, the stock culture was inoculated onto trypticase soy agar (TSA, Land Bridge Technology, Beijing, China) and cultured at 37°C overnight. Then a single colony was inoculated into TSB and the culture was incubated at 37°C, with shaking at 180 rpm, for 8 h. After activation, the cells from the culture were obtained by centrifugation (5000 × g, 4°C, 15 min). Precipitates were washed twice with phosphate-buffered saline (PBS, Land Bridge Technology, Beijing, China), and finally resuspended and adjusted to an OD_600nm_ = 0.5, with a cell concentration of 10^8^ CFU/mL. Finally, the bacterial suspensions were diluted to a concentration of 10^7^ CFU/mL prior to mature biofilm formation.

### Mature Biofilm Formation

The assay protocol for mature biofilm formation was based on that reported by Kim et al. (2006). Briefly, 30 mL of diluted bacterial suspension, prepared as described above, were inoculated into each centrifuge tube along with a stainless steel sheet. The sheets were cultured for 24 h at 4°C to ensure the adherence of *C. sakazakii*. After the adhesion stage, each inoculated sheet was gently rinsed for 15 s using 400 mL of sterile water, followed by rinsing for 5 s using 200 mL of sterile water. Then each sheet was placed in 30 mL of TSB and cultured for 48 h at 25°C. The biofilm was further confirmed to be mature using SEM, and was used in the following study.

### Population of Surviving Biofilm Cells After LED Illumination

Stainless steel sheets inoculated with mature biofilm were rinsed for 15 s using 400 mL of sterile water, then rinsed for 5 s using 200 mL of sterile water to remove loosely attached cells. For the LED-treated groups, the rinsed sheets in fixer were illuminated at 25, 10, and 4°C, respectively, while the rinsed sheets in fixer of the non-LED controls were placed in an incubator at 29.5, 15.5, and 12.5°C, protected from the light. At the specified times (0, 30, 60, 120, and 240 min), the sheets were transferred into 50 mL centrifuge tubes containing 3 g of glass beads (425–600 μm; Sigma-Aldrich, St. Louis, MO, USA) and 30 mL PBS (Kim et al., 2019). After vortexing for 5 min, the detached bacterial suspension was serially diluted in PBS, then 0.1 mL was spread-plated onto TSA plates and incubated at 37°C for 24 h. Survival cell counts were expressed as log CFU/cm^2^.

### Confocal Laser Scanning Microscopy (CLSM) Observations

CLSM observations were conducted as previously reported (Kang et al., 2018). After incubation, biofilms were illuminated at 25, 10, and 4°C for 2 h. Then sterile water was used to remove planktonic cells. SYTO 9 was used to stain the biofilm for 15 min at room temperature, protected from the light. After staining, the sheets were rinsed three times with sterile deionized water to remove excess stain. A Nikon A1 microscope (Nikon, Tokyo, Japan) was then used to obtain CLSM images using a 10× objective lens. CLSM images of non-LED-treated biofilms were also obtained as a control to observe the damage caused by LED exposure.

### Scanning Electron Microscopy (SEM) Observations

SEM imaging was performed to obtain a deeper understanding of the structure damage caused to *C. sakazakii* biofilm by exposure to the 405 nm LED, based on the method reported by Tong et al. (2018), with little modification. LED-illuminated and non-illuminated biofilms incubated at 25, 10, and 4°C for 2 h were fixed overnight with 2.5% glutaraldehyde at 4°C. The biofilms were then washed with sterile water and PBS, and soaked in 1% (v/v) osmic acid for 5 h, followed by gradient elution with 30, 50, 70, 80, 90, and 100% ethanol for 10 min. Finally, the dehydrated biofilms were splutter-coated with a thin layer of gold and examined under a S-4800 scanning electron microscope (Hitachi, Tokyo, Japan) at 4000× magnification.

### Effect of LED on Glycoconjugates in the Biofilm

The distribution of glycoconjugates within the biofilm matrix was analyzed according to a protocol described by Quilès et al. (2012). LED-illuminated and non-illuminated biofilms incubated at 25, 10, and 4°C for 2 h were stained with concanavalin A (Con-A) conjugated to fluorescein (excitation/emission: 494/518 nm, Invitrogen/Molecular Probes, Eugene, OR, USA) for 15 min at room temperature in the dark. Then, the plates were rinsed three times with sterile water to remove excess stain. Images of glycoconjugates were obtained with a Nikon A1 microscope (Nikon, Tokyo, Japan) using a 10× objective lens.

### Attenuated Total Reflection Fourier-Transformed Infrared Spectroscopy (ATR-FTIR)

Illuminated and non-illuminated biofilms incubated at 25, 10, and 4°C for 2 h were rinsed three times with 0.85% NaCl solution to remove loosely adhered thallus, and were then air-dried at room temperature for later use, according to a method described by Wang et al. (2018). The parameters of the ATR-FTIR spectrometer (NEXUS 670, Thermo Nicolet, USA) with 2 cm^−1^ resolution and 128 scans were set, and signals in the range of 2,000 to 800 cm^−1^ were captured. Sterile stainless steel was used to deduct the spectral background and OMNIC 8.2 software was used for data analysis.

### Effect of the LED on the Transcription of Genes Involved in *C. sakazakii* Biofilm Formation

To describe transcription level changes of genes involved in *C. sakazakii* biofilm formation after exposure to LED, RT-qPCR analysis was used according to previous reports with minor modifications (Abdallah et al., 2015). After incubation, LED-illuminated and non-LED-treated biofilm cells incubated at 25°C for 2 h, were detached by vortexing for 5 min. Then the bacterial sediment was obtained by centrifugation at 4,500 × g for 10 min (20°C) and washed twice with PBS. Total RNA extraction from the bacterial sediment was performed using the RNAprep Pure Bacteria Kit (Tiangeng, Beijing, China). RNA was reverse transcribed into cDNA using the PrimeScript™ RT Master Mix (TaKaRa, Beijing, China), according to the manufacturer's instructions. Real-time PCR was conducted in a 25-μL system using TB Green™ *Premix Ex Taq*™ II (TaKaRa, Beijing, China). The IQ5 system (Bio-Rad Laboratories, Hercules, CA, USA) was used to run the samples. The PCR conditions were: 1 cycle at 95°C for 30 s, followed by 40 cycles consisting of 95°C for 5 s and 60°C for 30 s, and dissociation steps of 95°C for 15 s and 60°C for 30 s. Primer sequences and PCR parameters for detecting genes related to biofilm formation (*bcsA, bcsG, flgJ, motA, motB, luxR, fliD*, and *flhD*) are listed in Table 1. The 2^−ΔΔCT^ method was used to express changes at the transcript level caused by LED illumination (Livak and Schmittgen, 2001). For *C. sakazakii*, the levels of transcription of biofilm-forming genes in LED-illuminated cells were calculated and compared with the transcription levels in non-illuminated biofilm cells.

**Table 1:** RT-qPCR primer sequences.

**Gene**	**Primer sequences (5**′**-3**′**)**
16SrRNA	F: AACCCTTATCCTTTGTTGCCA
	R: CGGACTACGACGCACTTTATG
*bcsA*	F: AAGAAGAGTACGTGGACTGGGTGA
	R: CGCCGAGGATAATCAGGTTGTAG
*bcsG*	F: GACGGGCTATCTGAATTTCCAC
	R: GCCAGGTATCATGCCAGAACA
*flgJ*	F: TCAGGTGCCGATGAAGTTTG
	R: GCCCTTTCCAGGACGATGT
*motA*	F: CGTGCTTTGGACACCATTT
	R: TCTCGTTTTCTTCCCTTTTCC
*motB*	F: TTCTGTTGCCTCCAGTTC
	R: CTCTTGTTCGTTGCTTCTTTC
*luxR*	F: AGCCATATGGATAGCGACATAGAG
	R: GCCGGATCCCTATTGGGCGAAAAG
*fliD*	F: ATCGAGATCGAGCGTTCCAC
	R: CGCCCTTATCAACTTTGACGTATT
*flhD*	F: GACTCTGCCGCAAATGGTG
	R: CCTTTTCTTCCTGGCGACG

### Changes in Sensitivity of LED-Illuminated *C. sakazakii* Biofilm Cells to Disinfectants

The changes in biofilm sensitivity to disinfectants were measured according to a previously reported method with minor modifications (Kang et al., 2018). At 2 and 4 h, illuminated and non-illuminated biofilms at 25, 10, and 4°C were carefully immersed in 50 mL centrifuge tubes containing a solution of CHX (30 mL, 100 ppm) and CTPC (30 mL, 100 ppm) for up to 15 min. They were then immediately transferred into 15 mL neutralization solution (Tween 80 30 g/L, saponin 30 g/L, sodium thiosulfate 5 g/L, L-histidine 1 g/L, lecithin 30 g/L, and TSB 9.5 g/L) to stop the activity of the disinfectant. After neutralization, each sample was transferred into a 50 mL centrifuge tube containing 3 g of glass beads (425–600 μm; Sigma-Aldrich, St. Louis, MO, USA) and 30 mL PBS, then vortexed for 5 min as described above. The detached bacterial suspension was serially diluted in PBS, then 0.1 mL of the diluted solutions was plated onto TSA plates and cultured at 37°C to determine the cell count. The difference between non-illuminated and 405-nm LED-illuminated *C. sakazakii* biofilm cell populations after disinfectants treatment was expressed as log reduction CFU/cm^2^.

### Statistical Analysis

Each treatment was replicated three times. Statistical analyses were performed using SPSS software (Version 18.0; SPSS, Inc., Chicago, IL, USA) and Tukey's test for average separation on an entirely randomized design with six sets of data. The data are presented as the mean ± *SD* (*n* = 3), and differences between the means were tested by one-way ANOVA or a Student's *t*-test. Differences with *P*-values of < 0.05 were considered statistically significant.

## Results

### Population of Viable Biofilm Cells After LED Illumination

**Figure 1:**
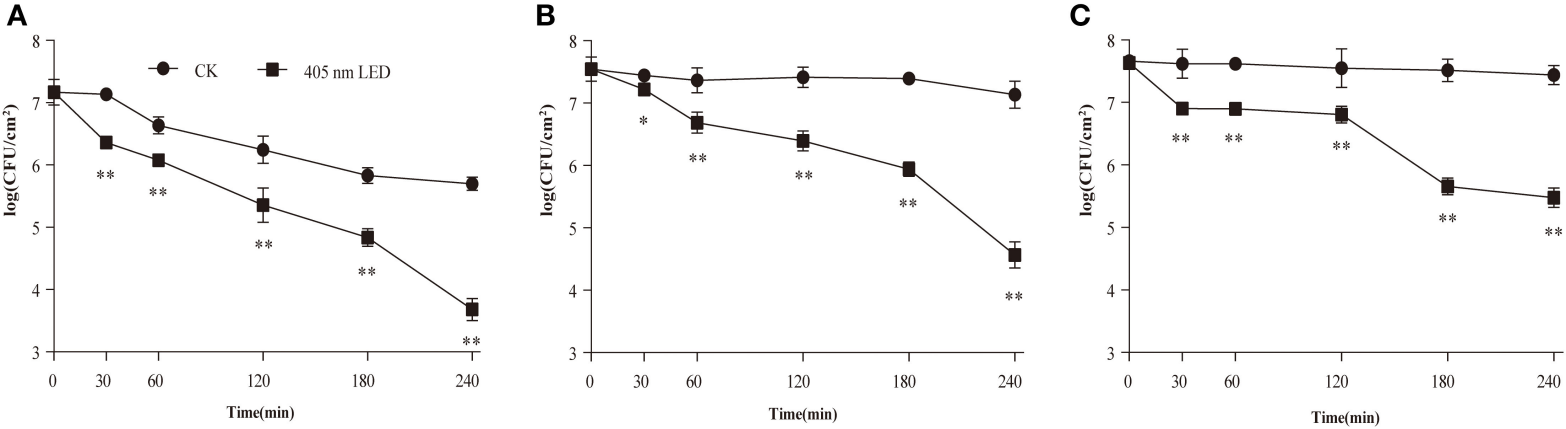
Inhibition of *C. sakazakii* biofilm cells on stainless steel by 405 nm LED illumination at 25°C **(A)**, 10°C **(B)**, and 4°C **(C)**. The errors bars indicate the standard error of the mean. *P < 0.05; **P < 0.01 vs. the control.

The effect of 405-nm LED illumination on viable cells in the mature *C. sakazakii* biofilm was determined (Figure 1). The population of non-illuminated biofilm cells remained relatively stable during storage at 10 and 4°C, showing a 0.4 log (Figure 1B) and 0.2 log (Figure 1C) reduction after 4 h, but was significantly decreased at 25°C, showing a 1.5 log (Figure 1A) reduction. After LED treatment for 4 h, all of the test groups showed a significant reduction in the biofilm cell population compared with the controls, with 2.0 log, 2.5 log and 2.0 log reductions at 25°C (Figure 1A), 10°C (Figure 1B), and 4°C (Figure 1C), respectively.

### Confocal Laser Scanning Microscopy (CLSM)

**Figure 2:**
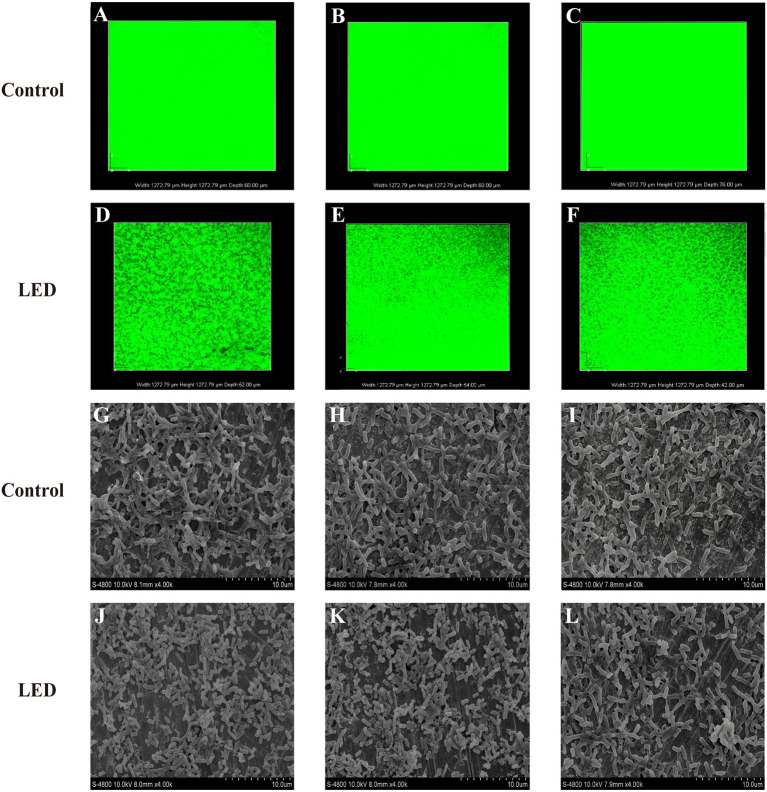
Confocal laser scanning microscopic images of *C. sakazakii* biofilms on stainless steel with or without 405 nm LED treatment for 2 h at 25°C **(A,D)**, 10°C **(B,E)**, and 4°C **(C,F)**. Scanning electron microscopic images of *C. sakazakii* biofilms on stainless steel with or without 405 nm LED treatment for 2 h at 25°C **(G,J)**, 10°C **(H,K)**, and 4°C **(I,L)**.

After LED illumination, damage to the conformational structure of the *C. sakazakii* biofilm was observed by CLSM (Figures 2A–F). For all of the biofilms in the non-illuminated groups, the whole field of vision showed green, indicating that the stereoscopic conformation of the biofilm was intact. However, after 2 h of illumination, the integrity and depth of the green fluorescence were significantly influenced by the more “porous” area, which indicated that the integrity of the biofilm stereoscopic conformation had been destroyed and the population of bacterial cells in the biofilm had decreased. This destruction was temperature-independent, revealing more “porous” areas and architectural damage at a set temperature of 25°C (Figures 2A,D) than at 10°C (Figures 2B,E) or 4°C (Figures 2C,F).

### Scanning Electron Microscopy (SEM) Imaging

To confirm the effects of LED on *C. sakazakii* biofilm in greater detail, images of inoculated sheets were taken under a scanning electron microscope (Figures 2G–L). The typical structure of mature biofilm was observed in the control groups, characterized by a complex three-dimensional structure formed by many cell colonies integrated by a network of extracellular matrix. By contrast, in the LED-treated groups, significant removal of the network of extracellular matrix and destruction of the dense three-dimensional structure were observed, resulting in biofilms of reduced thickness and separate cells without aggregation. Moreover, when the illumination temperature increased from 4°C (Figures 2I,L) and 10°C (Figures 2H,K) to 25°C (Figures 2G,J), the network of extracellular matrix and the three-dimensional structure of the biofilm gradually disappeared.

### Effect of LED Illumination on the Glycoconjugates in the Biofilm

**Figure 3:**
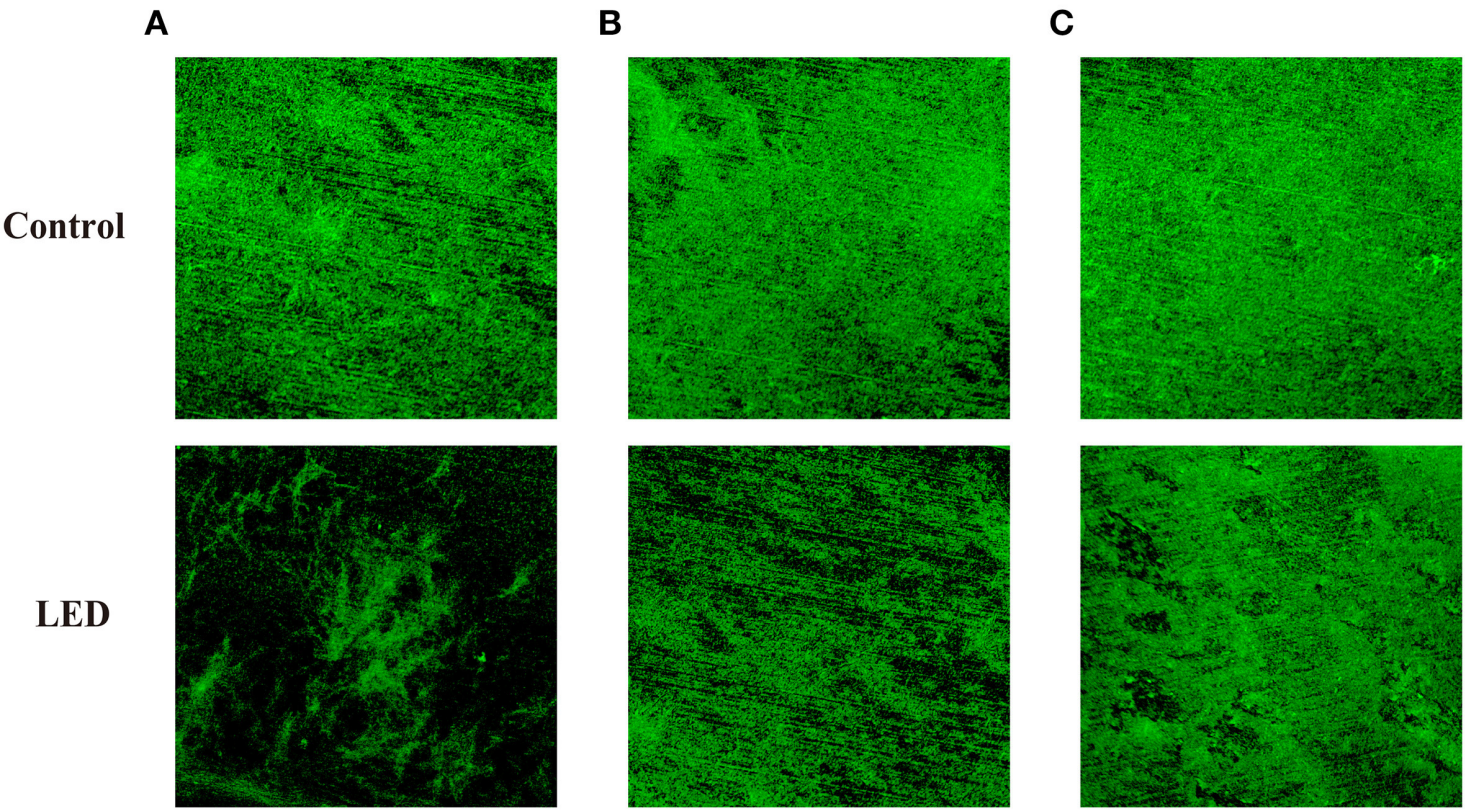
Glycoconjugates in the EPS of *C. sakazakii* biofilms formed on stainless steel after non-LED treatment and LED treatment for 2 h at 25°C **(A)**, 10°C **(B)**, and 4°C **(C)**.

**Figure 4:**
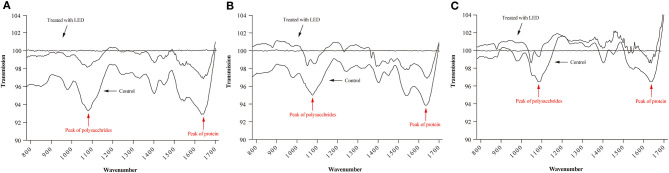
ATR-FTIR spectra of *C. sakazakii* biofilms formed on stainless steel after non-LED treatment and LED treatment for 2 h at 25°C **(A)**, 10°C **(B)**, and 4°C **(C)**.

ConA-fluorescein was used to determine the effects of LED illumination on the glycoconjugates in the EPS of *C. sakazakii* biofilm (Figure 3). Changes to the concentration and thickness of glycoconjugates in the biofilm were evident under the microscope. In contrast to the control biofilm that appeared well established in structure and contained glycoconjugates of the correct thickness, the LED-treated biofilm appeared damaged in structure with a lower concentration of glycoconjugates that were thinner, which supported the findings of CLSM and SEM. The damage to glycoconjugates caused by LED illumination was influenced by temperature, which was also in accordance with the CLSM and SEM observations, with a greater eliminating effect at 25°C (Figure 3A) than at 10°C (Figure 3B) and 4°C (Figure 3C).

### Effect of LED Illumination on Biofilm Composition

The ATR-FTIR spectra of LED-illuminated biofilms and non-illuminated biofilms were determined directly on stainless steel sheets at 25, 10, and 4°C (Figure 4). According to the functional groups associated with major bands in the ATR-FTIR spectra the spectral peaks at 1,084 and 1,056 cm^−1^ were assigned to polysaccharides and the peak at 1,647 cm^−1^ related to a specific protein peak. After LED illumination, the spectral peaks at 1,084, 1,056, and 1,647 cm^−1^ increased, which indicated the removal of polysaccharides and protein after LED illumination at 25, 10, and 4°C. These results showed that LED illumination caused significant removal of representative components of the biofilm, with the greatest eliminating effect observed at 25°C (Figure 4A), followed by 10°C (Figure 4B) and 4°C (Figure 4C).

### Effect of LED Illumination on the Transcription Level of Genes Involved in *C. sakazakii* Biofilm Formation

**Figure 5:**
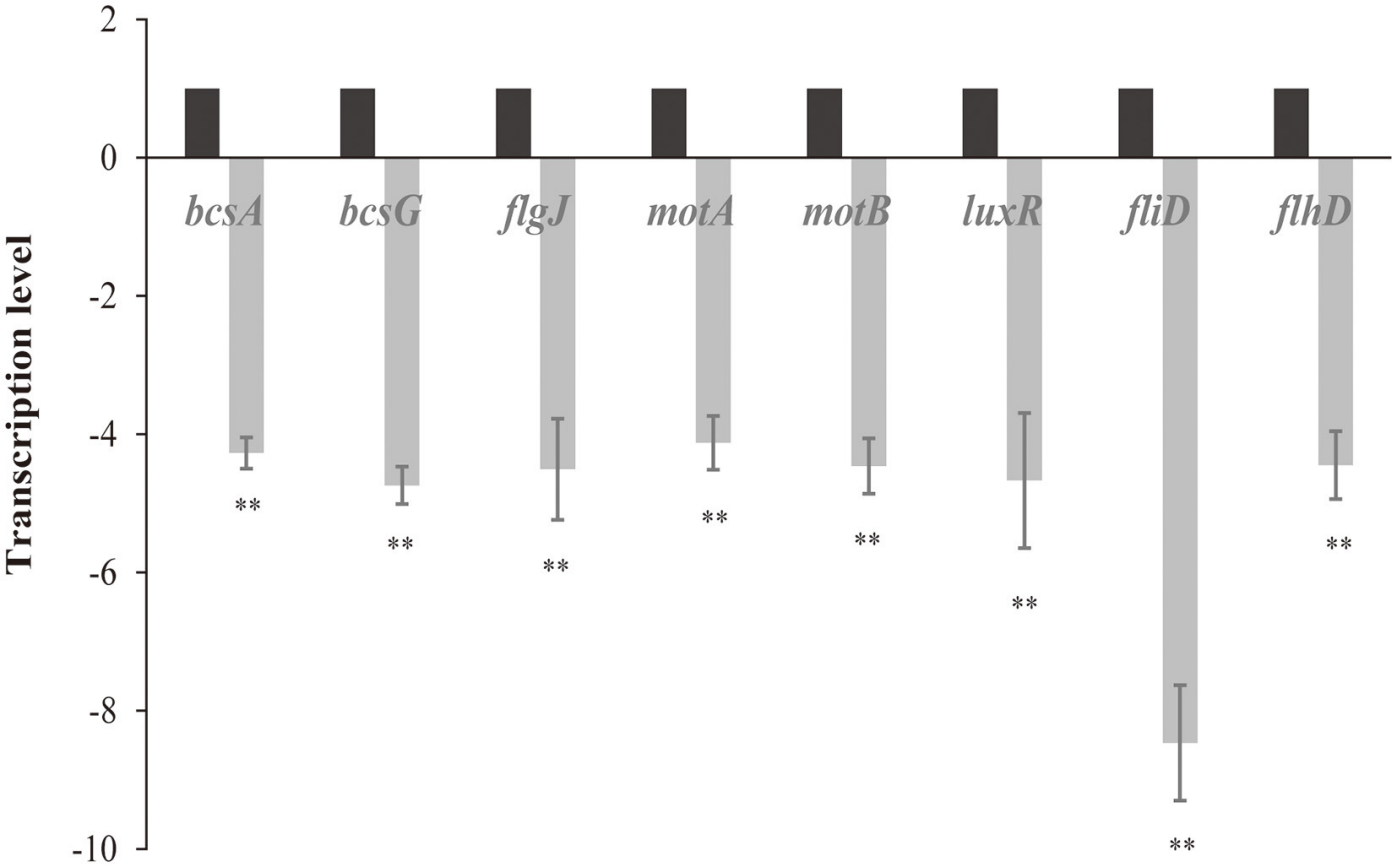
Transcription levels of genes involved in the formation of *C. sakazakii* biofilms after non-LED treatment and LED treatment for 2 h at 25°C. Bars indicate means ± the standard deviation. **P < 0.01.

The levels of transcription of genes related to *C. sakazakii* biofilm formation (*bcsA, bcsG, flgJ, motA, motB, luxR, fliD*, and *flhD*) under non-LED treatment and LED-illuminated treatment are shown in Figure 5. In the LED-illuminated groups, a significant reduction in the transcript level of all eight genes related to biofilm formation was observed (*P* < 0.01), with the greatest difference observed for the *fliD* gene.

### Sensitivity Changes of LED-Illuminated *C. sakazakii* Biofilm Cells to Disinfectants

**Figure 6:**
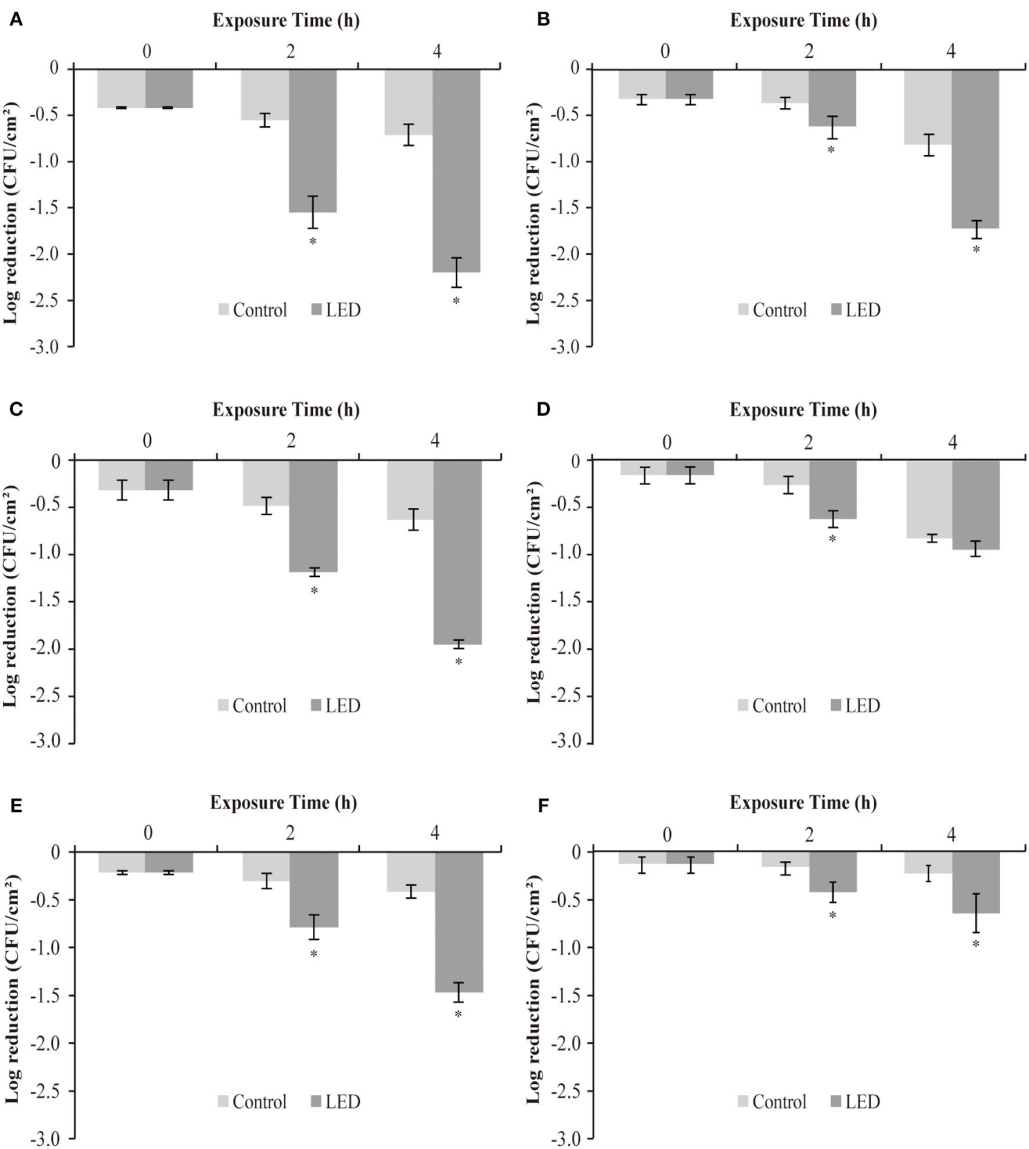
Log reduction between non-illuminated and 405-nm LED-illuminated *C. sakazakii* biofilm cells on stainless steel at 25°C **(A,B)**, 10°C **(C,D)**, and 4°C **(E,F)**, also treated with 100 ppm CHX **(A,C,E)** and CTPC **(B,D,F)** for 15 s. The errors bars indicate the standard error of the mean. The asterisk (*) indicates that the difference between non-illuminated control and illuminated biofilm cells population is significant (*P* < 0.05).

The effect of LED illumination on the sensitivity of *C. sakazakii* biofilm cells to disinfectants (CTPC and CHX) at 25, 10, and 4°C was determined (Figure 6). In the CHX-treated groups, after LED illumination for 2 h, reductions of 1.0 log, 0.7 log and 0.5 log between the LED-illuminated and non-illuminated groups at 25°C (Figure 6A), 10°C (Figure 6C), and 4°C (Figure 6E) were determined, respectively. After LED illumination for 4 h, reductions of 1.5 log (Figure 6A), 1.3 log (Figure 6C), and 1.1 log (Figure 6E) were observed, respectively. The results indicated that LED illumination significantly increase the sensitivity of *C. sakazakii* biofilm cells to CHX treatment, and stronger sensitivity changes were observed at 25°C.

The observations in groups treated with CTPC were a little different, showing reductions of 0.3 log, 0.4 log and 0.3 log between LED-illuminated and non-illuminated cells after 2 h, at 25°C (Figure 6B), 10°C (Figure 6D), and 4°C (Figure 6F), respectively. After LED illumination for 4 h, reductions of 0.9 log (Figure 6B), 0.1 log (Figure 6D), and 0.4 log (Figure 6F) were determined, respectively. The differences between the reductions in cell numbers between non-LED and LED treatment groups indicated that biofilm cells showed stronger sensitivity to CTPC after LED illumination, in which greater sensitivity changes were observed at 25°C.

## Discussion

*C. sakazakii* shows good tolerance to a variety of external stresses (Ling et al., 2020) and conventional antibiotics (Davies, 2003) as a result of its strong biofilm-forming ability, which reduces the effects of traditional decontamination methods. *C. sakazakii* has therefore become a major source of infant formula contamination (Bayoumi et al., 2012). On infection, *C. sakazakii* can cause bacteremia, meningitis, necrotic enteritis and other serious diseases in infants or adults with low immunity, and is therefore a cause of widespread concern (Abdesselam and Pagotto, 2014). A safe and effective new method for the removal of *C. sakazakii* biofilm would therefore be of great value. Studies have shown that LED illumination has an inactivation effect on a variety of pathogens in food (McKenzie et al., 2013; Ghate et al., 2019). In this study, we evaluated the inactivation effect of 405 nm LED illumination on the biofilm of *C. sakazakii* and compared the sensitivity of LED-illuminated and non-illuminated biofilm cells to disinfectant treatment.

Before determining the population of surviving biofilm cells after 405 nm LED illumination, the face temperature changes of the stainless steel sheets were monitored and recorded at 2-min intervals for 120 min during illumination (data not shown). For the three set temperatures (25, 10, and 4°C), the temperatures were maintained at 29.5, 15.5, and 12.5°C after 120 min. To compensate for this heating effect and to ascertain whether the anti-biofilm effect was only a result of photodynamic inactivation, non-illuminated control experiments were adjusted to 29.5, 15.5, and 12.5°C.

The population of surviving *C. sakazakii* biofilm cells after LED illumination was first determined in this study. The results showed that LED illumination was able to significantly reduce the population of surviving biofilm cells at 25, 10, and 4°C (Figure 1). Besides, previous reports have confirmed the mechanism of LED inactivation against planktonic pathogens. Ghate et al. (2013) and Kumar et al. (2016) provided evidence of the inactivation effect of LED illumination against planktonic *E. coli, Salmonella, Listeria*, and *S. aureus* based on the reduction in the cell population and reported that LED inactivation is a direct consequence of DNA and cell membrane function loss caused by ROS generated by LED light. Our study thus indicated that, although biofilm could protect a certain number of cells from external hazards, 405 nm LED illumination might still exert its inactivation efficacy against surviving *C. sakazakii* cells via the generation of ROS.

Interestingly, we found that the total reduction of viable biofilm cells between the LED-illuminated groups after 4 h and the controls at 0 h was dependent on temperature, with a 3.5-log (Figure 1A), 2.9-log (Figure 1B) and 2.2-log (Figure 1C) reduction after LED treatment at 25, 10, and 4°C, respectively. This may be because the effect of temperature on the reduction of biofilm cells, especially at 25°C, showed a 1.5-log (Figure 1A) reduction for non-illuminated biofilm cells after 4 h, enhancing the inactivation efficacy of 405 nm LED illumination against biofilm cells significantly. However, previous study has reported the opposite phenomenon, which showed that low temperatures could induce the increase the proportion of unsaturated fatty acids in bacteria (Luksiene, 2003). Unsaturated fatty acids are susceptible to ROS generated by cells, thus enhance the inactivation efficacy of LED and resulting in more cell death (Beales, 2004). Hence, the effects of temperature on the efficacy of LED illumination against *C. sakazakii* biofilm should be further confirmed in future studies.

An intact biofilm matrix is essential for biofilm to protect pathogen cells from death (Wang et al., 2018). In the current study, the integrity of the biofilm stereoscopic conformation of LED treatment groups was damaged compared with the control groups by observation of CLSM images (Figures 2A–F), and greater damage was observed at 25°C which was consistent with the results of cells population determination (Figures 2A,D). Similarly, Loo et al. (2016) observed that the integrity and thickness of the *S. aureus* biofilm was significantly reduced after the treatment with 50 μg/mL of silver nanoparticles, as evidenced by CLSM images. Wang et al. (2018) presented the CLSM images of *P. fluorescens* biofilm with a reduced biofilm matrix and killed cells, when treated with 200 mg/L sodium hypochlorite and 40 mg/L acidic electrolyzed water.

As the complement of CLSM, SEM images showed that 405 nm LED illumination caused significant destruction of the biofilm architecture (Figures 2G–L), especially at 25°C (Figures 2G,J), by destroying the complex three-dimensional structure formed by cell colonies integrated by the network of extracellular matrix. Previous reports also showed the similar destructions to biofilms caused by other antibiofilm strategies, which lead to the detachment of cells. Mamone et al. (2016) found that 5,10,15,20-tetrakis[4-(3-N,N-dimethylammoniumpropoxy)phenyl]porphyrin-mediated photodynamic inactivation could lead to the detachment of parts of the *S. aureus* biofilm and disruption of its architecture, as evidenced by SEM images. Di Poto et al. (2009) also provided SEM images and reported that photodynamic treatment combined with vancomycin caused a significant reduction in the matrix and the size of cell aggregates in *S. aureus* biofilm. Our study provided clear evidence of the reduction of *C. sakazakii* biofilm matrix as well as the destruction of architectural features of biofilm caused by LED illumination, thus making the structure of the entire biofilm less stable and the cells more detached. The current results indicate that the damaged biofilm structure might be caused by reduced cells population which confirmed above or the reduction of biofilm matrix.

To further evaluate the loss of biofilm matrix, ATR-FTIR and glycoconjugates observation were conducted in the current study. Figure 4 confirmed the removal of polysaccharide and protein by LED treatment, which were two representative components of the biofilm (Misba et al., 2017), and this effect was greatest at 25°C (Figure 4A). In similar studies, the removal of EPS compounds (mainly proteins and polysaccharides) from biofilms has been demonstrated by other types of treatment. Lee et al. (2017) reported that the combination of Cu (II) with norspermidine caused significant degradation of EPS (44 and 63% degradation of proteins and polysaccharides, respectively), by calculating the respective bioareas of proteins and polysaccharides using MetaMorph software. Misba et al. (2017) also reported that phenothiazinium dyes could decrease the EPS contents of *Enterococcus faecalis* and *Klebsiella pneumoniae* biofilms based on a Congo red binding assay.

The results of glycoconjugates observation confirmed that LED illumination could significantly reduce the concentration of glycoconjugates (Figure 3), especially at 25°C (Figure 3A). Paramanantham et al. (2018) reported a 54.93% reduction in the exopolysaccharide content of biofilm after treatment with an amino-functionalized mesoporus silica-rose Bengal nanoconjugate using a Congo red binding assay. The mechanism of action of LED illumination against several intracellular substances has been reported and is thought to involve ROS generated by cells in response to LED light that may react with DNA, proteins, lipids, and other components to produce cytotoxic effects (Luksiene, 2010). Combined the results of CLSM and SEM images, we hypothesize that accumulated ROS generated by LED illumination might also play an important role in the removal of protein and polysaccharide in *C. sakazakii* biofilm, resulting in the detached cells and damaged structure.

The RT-qPCR results showed that LED illumination significantly reduced the transcription level of genes related to *C. sakazakii* biofilm (Figure 5). Flagellum mediated motility has been reported to play an important role in initiation of biofilm formation through increasing the likelihood of bacterial interacts with the contacting surfaces as well as providing physical frames in biofilm matrix (Jung et al., 2019). The *motA, motB, fliD, flhD*, and *flgJ* genes observed in this study have been showed to be responsible for the synthesis process of flagellum that are involved in bacterial biofilm formation. FlhD is a specific activator of flagellin synthesis (Prüß et al., 2001) and FlgJ is a two-domain flagellar protein, with one domain involved in rod assembly and the other playing an enzymatic role as a muramidase (Li et al., 2012). FliD protein is an important protein for the *in vivo* colonization and the formation of functional flagella (Ratthawongjirakul et al., 2016). *motA, motB* genes are responsible for the synthesis of flagellin complexes which is important for the motility of bacteria (Kojima and Blair, 2004). Our results indicated that LED could regulate functions of *motA, motB, fliD, flhD*, and *flgJ*, controlling the synthesis of flagellum, thus influencing the bacterial motility and biofilm structure.

Quorum sensing is a cell-cell communication which bacteria use to utilize diverse signal recognition systems and subsequent regulatory mechanisms to achieve timely expression of EPS components during the specific stages of biofilm formation or under specific conditions (Waters et al., 2008). For the *luxR* determined in current study, previous study has reported that it encodes the LuxR-type regulators which is responsible for the regulation of quorum sensing protein synthesis then influence the establishment of biofilm (Hou et al., 2018). The results in current study indicated that LED illumination could control the synthesis of quorum sensing protein which are important for the timely expression of EPS, thereby affecting the biofilm-forming ability of *C. sakazakii* cells. Cellulose is a major component of biofilm matrix and may also act as an adhesion factor (El Hag et al., 2017). *bcsA* and *bcsG* genes are necessary to produce cellulose, and are involved in cell–cell aggregation and biofilm formation (Hu et al., 2015; Anderson et al., 2020). Our study suggested that LED may control the synthesis of cellulose and adhesion ability of *C. Sakazakii* cells by regulating the function of *bcsA* and *bcsG*, thus influencing the biofilm forming process.

Some biofilm cells possess a degree of resistance to disinfectants, which increases the difficulty of using disinfectants to eliminate biofilms (Simões et al., 2009). What's worse, the overuse of disinfectants may induce stronger resistance in pathogens and leave harmful residues to environment (Li et al., 2014). Therefore, in current study, we studied how 405 nm LED illumination would influence the resistance of biofilm cells to disinfectant treatment. Two food disinfectants (CTPC and CHX) were selected to determine the effect of LED treatment on the sensitivity of biofilm cells. CHX is a protein synthesis inhibitor (Barros et al., 2015) and CTPC is a quaternary ammonium salt that functions as a disinfectant. Both exert their antibacterial activity by denaturing proteins and enzymes, destroying cell membrane integrity and causing the leakage of cell contents (Imai et al., 2017). Figure 6 showed that the sensitivity of *C. sakazakii* biofilm cells to CTPC and CHX increased after exposure to the LED. Regarding biofilm resistance, Harper et al. (2019) provided evidence that the extracellular polymer substances generated by biofilms confer resistance to antimicrobial agents through electrostatic and steric interactions that hinder molecular diffusion. Our study further established that LED illumination may cause damage to the biofilm by denaturing the composition and architectural features of biofilm, reducing the resistance of the biofilm to disinfectants and allowing disinfectant molecules access into cells. In addition, the change in resistance was influenced by both the illumination temperature and the type of disinfectant. The results of current study indicated that 405 nm LED could be used in food industry to eliminate biofilms as the complement of disinfectants to reduce the dosage of disinfectants used and enhance the antibiofilm effect of disinfectants.

## Conclusion

In conclusion, 405-nm LED illumination was effective at inactivating mature *C. sakazakii* biofilm. Significant reduction of the population of viable biofilm cells was observed after LED illumination. Clear evidence of the structural damage caused by LED was provided by the images of CLSM, SEM and glycoconjugates, which showed the significant destruction of the architectural feature of LED-treated biofilms. Results of ATR-FTIR and RT-qPCR analysis revealed that LED could remove the biofilm compositions (mainly polysaccharide and protein) and influence genes function related to biofilm formation. In addition, the sensitivity of biofilm cells to disinfectants treatment was also observed to increase, enhancing the antibacterial effect of disinfectants against biofilm. Our findings suggested that 405-nm LED illumination has the potential to be administered directly as a new antibacterial method to inactivate *C. sakazakii* biofilm in various food processing applications, such as milk powder processing, packaging tank processing and the cleaning of household brewing utensils. In the future, studies are expected to evaluate LED inactivation efficacy against biofilms formed by multiple pathogens that attach to various contact surfaces, such as glass, silicone, and Teflon. The combined inactivation efficacy of LED illumination with photosensitizers should also be studied.

## Data Availability Statement

The raw data supporting the conclusions of this article will be made available by the authors, without undue reservation.

## Author Contributions

YH, QP, and CS conceived and designed the experiments. YH, RD, XZ, and JG performed the experiments. YH, QP, and DG analyzed the data. YY and SL contributed reagents/materials/analysis tools. YH and CS wrote the manuscript. All authors contributed to the article and approved the submitted version.

## Conflict of Interest

The authors declare that the research was conducted in the absence of any commercial or financial relationships that could be construed as a potential conflict of interest.

## References

[B1] Abdallah M. Khelissa O. Ibrahim A. Benoliel C. Heliot L. Dhulster P.. (2015). Impact of growth temperature and surface type on the resistance of *Pseudomonas aeruginosa* and *Staphylococcus aureus* biofilms to disinfectants. Int. J. Food Microbiol. 214, 38–47. 10.1016/j.ijfoodmicro.2015.07.022 26233298

[B2] Abdesselam K. Pagotto F. (2014). Bacteria: *Cronobacter* (*Enterobacter*) *sakazakii* and other *Cronobacter* spp. Encycl. Food Safety 1, 424–432. 10.1016/B978-0-12-378612-8.00097-419729216

[B3] Anderson A. C. Burnett A. J. N. Hiscock L. Maly K. E. Weadge J. T. (2020). The *Escherichia coli* cellulose synthase subunit G (BcsG) is a Zn^2+^-dependent phosphoethanolamine transferase. J. Biol. Chem. 295, 6225–6235. 10.1074/jbc.RA119.011668 32152228 PMC7196641

[B4] Barros J. Grenho L. Fernandes M. H. Manuel C. M. Melo L. F. Nunes O. C.. (2015). Anti-sessile bacterial and cytocompatibility properties of CHX-loaded nanohydroxyapatite. Colloid. Surf. B 130, 305–314. 10.1016/j.colsurfb.2015.04.034 25936560

[B5] Bayoumi M. A. Kamal R. M. Abd El Aal S. F. Awad E. I. (2012). Assessment of a regulatory sanitization process in Egyptian dairy plants in regard to the adherence of some food-borne pathogens and their biofilms. Int. J. Food Microbiol. 158, 225–231. 10.1016/j.ijfoodmicro.2012.07.021 22884171

[B6] Beales N. (2004). Adaptation of microorganisms to cold temperatures, weak acid preservatives, low pH, and osmotic stress: a review. Compr. Rev. Food Sci. F 3, 1–20. 10.1111/j.1541-4337.2004.tb00057.x 33430556

[B7] Beuchat L. R. Kim H. Gurtler J. B. Lin L. C. Ryu J. H. Richards G. M. (2009). *Cronobacter sakazakii* in foods and factors affecting its survival, growth, and inactivation. Int. J. Food Microbiol. 136, 204–213. 10.1016/j.ijfoodmicro.2009.02.029 19346021

[B8] da Silva E. P. De Martinis E. C. P. (2013). Current knowledge and perspectives on biofilm formation: the case of *Listeria monocytogenes*. Appl. Microbiol. Biotechnol. 97, 957–968. 10.1007/s00253-012-4611-1 23233205

[B9] Davies D. (2003). Understanding biofilm resistance to antibacterial agents. Nat. Rev. Drug Discov. 2, 114–122. 10.1038/nrd1008 12563302

[B10] Di Poto A. Sbarra M. S. Provenza G. Visai L. Speziale P. (2009). The effect of photodynamic treatment combined with antibiotic action or host defence mechanisms on *Staphylococcus aureus* biofilms. Biomaterials 30, 3158–3166. 10.1016/j.biomaterials.2009.02.038 19329182

[B11] El Hag M. Feng Z. Su Y. Wang X. Yassin A. Chen S.. (2017). Contribution of the *csgA* and *bcsA* genes to *Salmonella enterica* serovar Pullorum biofilm formation and virulence. Avian Pathol. 46, 541–547. 10.1080/03079457.2017.1324198 28470089

[B12] Ghate V. Zelinger E. Shoyhet H. Hayouka Z. (2019). Inactivation of *Listeria monocytogenes* on paperboard, a food packaging material, using 410 nm light emitting diodes. Food Control 96, 281–290. 10.1016/j.foodcont.2018.09.026

[B13] Ghate V. S. Ng K. S. Zhou W. Yang H. Khoo G. H. Yoon W. B.. (2013). Antibacterial effect of light emitting diodes of visible wavelengths on selected foodborne pathogens at different illumination temperatures. Int. J. Food Microbiol. 166, 399–406. 10.1016/j.ijfoodmicro.2013.07.018 24026011

[B14] Ha J. W. Kang D. H. (2014). Synergistic bactericidal effect of simultaneous near-infrared radiant heating and UV radiation against *Cronobacter sakazakii* in powdered infant formula. Appl. Environ. Microbiol. 80, 1858–1863. 10.1128/AEM.03825-13 24413596 PMC3957628

[B15] Harouna S. Carramiñana J. J. Navarro F. Pérez M. D. Calvo M. Sánchez L. (2015). Antibacterial activity of bovine milk lactoferrin on the emerging foodborne pathogen *Cronobacter sakazakii*: effect of media and heat treatment. Food Control 47, 520–525. 10.1016/j.foodcont.2014.07.061

[B16] Harper R. A. Carpenter G. H. Proctor G. B. Harvey R. D. Gambogi R. J. Geonnotti A. R.. (2019). Diminishing biofilm resistance to antimicrobial nanomaterials through electrolyte screening of electrostatic interactions. Colloid. Surf. B 173, 392–399. 10.1016/j.colsurfb.2018.09.018 30317126

[B17] Hou H. Wang Y. Zhang G. Zhu Y. Xu L. Hao H.. (2018). Effects of sulfide flavors on AHL-Mediated quorum sensing and biofilm formation of *Hafnia alvei*. J. Food Sci. 83, 2550–2559. 10.1111/1750-3841.14345 30221799

[B18] Hu L. Grim C. J. Franco A. A. Jarvis K. G. Sathyamoorthy V. Kothary M. H.. (2015). Analysis of the cellulose synthase operon genes, *bcsA, bcsB*, and *bcsC* in *Cronobacter* species: prevalence among species and their roles in biofilm formation and cell–cell aggregation. Food Microbiol. 52, 97–105. 10.1016/j.fm.2015.07.007 26338122

[B19] Imai H. Kita F. Ikesugi S. Abe M. Sogabe S. Nishimura-Danjobara Y.. (2017). Cetylpyridinium chloride at sublethal levels increases the susceptibility of rat thymic lymphocytes to oxidative stress. Chemosphere 170, 118–123. 10.1016/j.chemosphere.2016.12.023 27984775

[B20] Jung Y. C. Lee M. A. Lee K. H. (2019). Role of flagellin-homologous proteins in biofilm formation by pathogenic *Vibrio* species. mBio 10, e01793–e01719. 10.1128/mBio.01793-19 31409687 PMC6692518

[B21] Kang J. Liu L. Liu M. Wu X. Li J. (2018). Antibacterial activity of gallic acid against *Shigella flexneri* and its effect on biofilm formation by repressing *mdoH* gene expression. Food Control 94, 147–154. 10.1016/j.foodcont.2018.07.011

[B22] Kim H. Ryu J. H. Beuchat L. R. (2006). Attachment of and biofilm formation by *Enterobacter sakazakii* on stainless steel and enteral feeding tubes. Appl. Environ. Microbiol. 72:5846. 10.1128/AEM.00654-06 16957203 PMC1563662

[B23] Kim Y. Kim H. Beuchat L. R. Ryu J. H. (2019). Inhibition of *Listeria monocytogenes* using biofilms of non-pathogenic soil bacteria (*Streptomyces* spp.) on stainless steel under desiccated condition. Food Microbiol. 79, 61–65. 10.1016/j.fm.2018.11.007 30621876

[B24] Kojima S. Blair D. F. (2004). Solubilization and purification of the MotA/MotB complex of *Escherichia coli*. Biochemistry 43:26. 10.1021/bi035405l 14705928

[B25] Krames M. Grandjean N. (2017). Light-emitting diode technology and applications: introduction. Photonics Res. 5, 6–7. 10.1364/PRJ.5.00LED1

[B26] Kumar A. Ghate V. Kim M. J. Zhou W. Khoo G. H. Yuk H. G. (2016). Antibacterial efficacy of 405, 460, and 520 nm light emitting diodes on *Lactobacillus plantarum, Staphylococcus aureus* and *Vibrio parahaemolyticus*. J. Appl. Microbiol. 120, 49–56. 10.1111/jam.12975 26481103

[B27] Kuo L. S. Wang B. J. He Y. S. Weng Y. M. (2013). The effects of ultraviolet light irradiation and drying treatments on the survival of *Cronobacter* spp. (*Enterobacter sakazakii*) on the surfaces of stainless steel, Teflon and glass. Food Control 30, 106–110. 10.1016/j.foodcont.2012.06.015

[B28] Lee H.-J. Seo J. Kim M. S. Lee C. (2017). Inactivation of biofilms on RO membranes by copper ion in combination with norspermidine. Desalination, 424, 95–101. 10.1016/j.desal.2017.09.034

[B29] Li R. Kuda T. Yano T. (2014). Effect of food residues on efficiency of surfactant disinfectants against food related pathogens adhered on polystyrene and ceramic surfaces. LWT Food Sci. Technol. 57, 200–206. 10.1016/j.lwt.2013.11.018

[B30] Li X. Xu J. Xie Y. Qiu Y. Fu S. Yuan X.. (2012). Vaccination with recombinant flagellar proteins *FlgJ* and *FliN* induce protection against *Brucella abortus* 544 infection in BALB/c mice. Vet. Microbiol. 161, 137–144. 10.1016/j.vetmic.2012.07.016 22854331

[B31] Ling N. Forsythe S. Wu Q. Ding Y. Zhang J. Zeng H. (2020). Insights into *Cronobacter sakazakii* biofilm formation and control strategies in the food industry. Engineering, 6, 393–405. 10.1016/j.eng.2020.02.007

[B32] Livak K. J. Schmittgen T. D. (2001). Analysis of relative gene expression data using real-time quantitative PCR and the 2^−ΔΔCT^ method. Methods 25, 402–408. 10.1006/meth.2001.1262 11846609

[B33] Loo C. Y. Rohanizadeh R. Young P. M. Traini D. Cavaliere R. Whitchurch C. B.. (2016). Combination of silver nanoparticles and curcumin nanoparticles for enhanced anti-biofilm activities. J. Agric. Food Chem. 64, 2513–2522. 10.1021/acs.jafc.5b04559 26595817

[B34] Luksiene Z. (2003). Photodynamic therapy: mechanism of action and ways to improve the efficiency of treatment. Medicina 39, 1137–1150. 10.1590/S0100-40422002000500016 14704501

[B35] Luksiene Z. (2010). Photosensitization and food safety. Chem. Technol. 4, 62–65. 10.1081/E-EBAF-120045486

[B36] Mamone L. Ferreyra D. D. Gándara L. Di Venosa G. Vallecorsa P. Sáenz D.. (2016). Photodynamic inactivation of planktonic and biofilm growing bacteria mediated by a meso-substituted porphyrin bearing four basic amino groups. J. Photochem. Photobiol. B 161, 222–229. 10.1016/j.jphotobiol.2016.05.026 27285813

[B37] McKenzie K. Maclean M. Timoshkin I. V. Endarko E. MacGregor S. J. Anderson J. G. (2013). Photoinactivation of bacteria attached to glass and acrylic surfaces by 405 nm light: potential application for biofilm decontamination. Photochem. Photobiol. 89, 927–935. 10.1111/php.12077 23550978

[B38] Misba L. Zaidi S. Khan A. U. (2017). A comparison of antibacterial and antibiofilm efficacy of phenothiazinium dyes between Gram positive and Gram negative bacterial biofilm. Photodiagn. Photodyn. 18, 24–33. 10.1016/j.pdpdt.2017.01.177 28119141

[B39] Moloney M. G. (2016). Natural products as a source for novel antibiotics. Trends Pharmacol. Sci. 37, 689–701. 10.1016/j.tips.2016.05.001 27267698

[B40] Paramanantham P. Antony A. P. Sruthil Lal S. B. Sharan A. Syed A. Ahmed M. (2018). Antimicrobial photodynamic inactivation of fungal biofilm using amino functionalized mesoporus silica-rose bengal nanoconjugate against *Candida albicans*. Sci. Afr. 1:e00007 10.1016/j.sciaf.2018.e00007

[B41] Park J. H. Lee J. H. Cho M. H. Herzberg M. Lee J. (2012). Acceleration of protease effect on *Staphylococcus aureus* biofilm dispersal. FEMS Microbiol. Lett. 335, 31–38. 10.1111/j.1574-6968.2012.02635.x 22784033

[B42] Prüß B. M. Liu X. Hendrickson W. Matsumura P. (2001). *FlhD*/*FlhC*-regulated promoters analyzed by gene array and *lacZ* gene fusions. FEMS Microbiol. Lett. 197, 91–97. 10.1016/S0378-1097(01)00092-1 11287152

[B43] Quilès F. Polyakov P. Humbert F. Francius G. J. B. (2012). Production of extracellular glycogen by *Pseudomonas fluorescens*: spectroscopic evidence and conformational analysis by biomolecular recognition. Biomacromolecules 13:2118. 10.1021/bm300497c 22686500

[B44] Quishida C. C. C. De Oliveira Mima E. G. Jorge J. H. Vergani C. E. Bagnato V. S. Pavarina A. C. (2016). Photodynamic inactivation of a multispecies biofilm using curcumin and LED light. Lasers Med. Sci. 31, 997–1009. 10.1007/s10103-016-1942-7 27126412

[B45] Ratthawongjirakul P. Thongkerd V. Chaicumpa W. (2016). The impacts of a *fliD* mutation on the biofilm formation of *Helicobacter pylori*. Asian Pac. J. Trop. Bio. 6, 1008–1014. 10.1016/j.apjtb.2016.10.005

[B46] Reich F. König R. von Wiese W. Klein G. (2010). Prevalence of *Cronobacter* spp. in a powdered infant formula processing environment. Int. J. Food Microbiol. 140, 214–217. 10.1016/j.ijfoodmicro.2010.03.031 20409601

[B47] Simões M. Bennett R. N. Rosa E. A. S. (2009). Understanding antimicrobial activities of phytochemicals against multidrug resistant bacteria and biofilms. Nat. Prod. Rep. 26, 746–757. 10.1039/b821648g 19471683

[B48] Simões M. Simões L. C. Vieira M. J. (2010). A review of current and emergent biofilm control strategies. LWT Food Sci. Technol. 43, 573–583. 10.1016/j.lwt.2009.12.008

[B49] Stephan R. Grim C. J. Gopinath G. R. Mammel M. K. Sathyamoorthy V. Trach L. H.. (2014). Re-examination of the taxonomic status of *Enterobacter helveticus, Enterobacter pulveris* and *Enterobacter turicensis* as members of the genus *Cronobacter* and their reclassification in the genera *Franconibacter* gen. nov. and *Siccibacter* gen. nov. as *Franconibacter helveticus* comb. nov., *Franconibacter pulveris* comb. nov., and *Siccibacter turicensis* comb. nov., respectively. Int. J. Food Microbiol. 64, 3402–3410. 10.1099/ijs.0.059832-0 25028159 PMC4179279

[B50] Tong L. Jiao R. Zhang X. Ou D. Wang Y. Zhang J.. (2018). Inhibitory effects of chitosan on *Cronobacter malonaticus* cells and biofilm formation. LWT-Food Sci. Technol. 97, 302–307. 10.1016/j.lwt.2018.07.00829102134

[B51] Wang H. Cai L. Li Y. Xu X. Zhou G. (2018). Biofilm formation by meat-borne *Pseudomonas fluorescens* on stainless steel and its resistance to disinfectants. Food Control 91, 397–403. 10.1016/j.foodcont.2018.04.035

[B52] Waters C. M. Lu W. Rabinowitz J. D. Bassler B. L. (2008). Quorum sensing controls biofilm formation in *Vibrio cholerae* through modulation of cyclic di-GMP levels and repression of *vpsT*. J. Bacteriol. 190, 2527–2536. 10.1128/JB.01756-07 18223081 PMC2293178

[B53] Yan Q. Q. Condell O. Power K. Butler F. Tall B. D. Fanning S. (2012). *Cronobacter* species (formerly known as *Enterobacter sakazakii*) in powdered infant formula: a review of our current understanding of the biology of this bacterium. J. Appl. Microbiol. 113, 1–15. 10.1111/j.1365-2672.2012.05281.x 22420458

[B54] Yang Y. Kumar A. Zheng Q. Yuk H. G. (2015). Preacclimation alters *Salmonella Enteritidis* surface properties and its initial attachment to food contact surfaces. Colloid. Surf. B. 128, 577–585. 10.1016/j.colsurfb.2015.03.011 25800356

[B55] Ye Y. Li H. Wu Q. Zhang J. Lu Y. (2014). The *Cronobacter* spp. in milk and dairy products: detection and typing. Int. J. Dairy Technol. 67, 167–175. 10.1111/1471-0307.12111

