# Supplementary figures and images for: Inactivation Efficacy of 405 nm LED Against Cronobacter sakazakii Biofilm
Source: Front Microbiol. 2020 Nov 27;11:610077. doi: 10.3389/fmicb.2020.610077 (PMC7728857; doi:10.3389/fmicb.2020.610077)

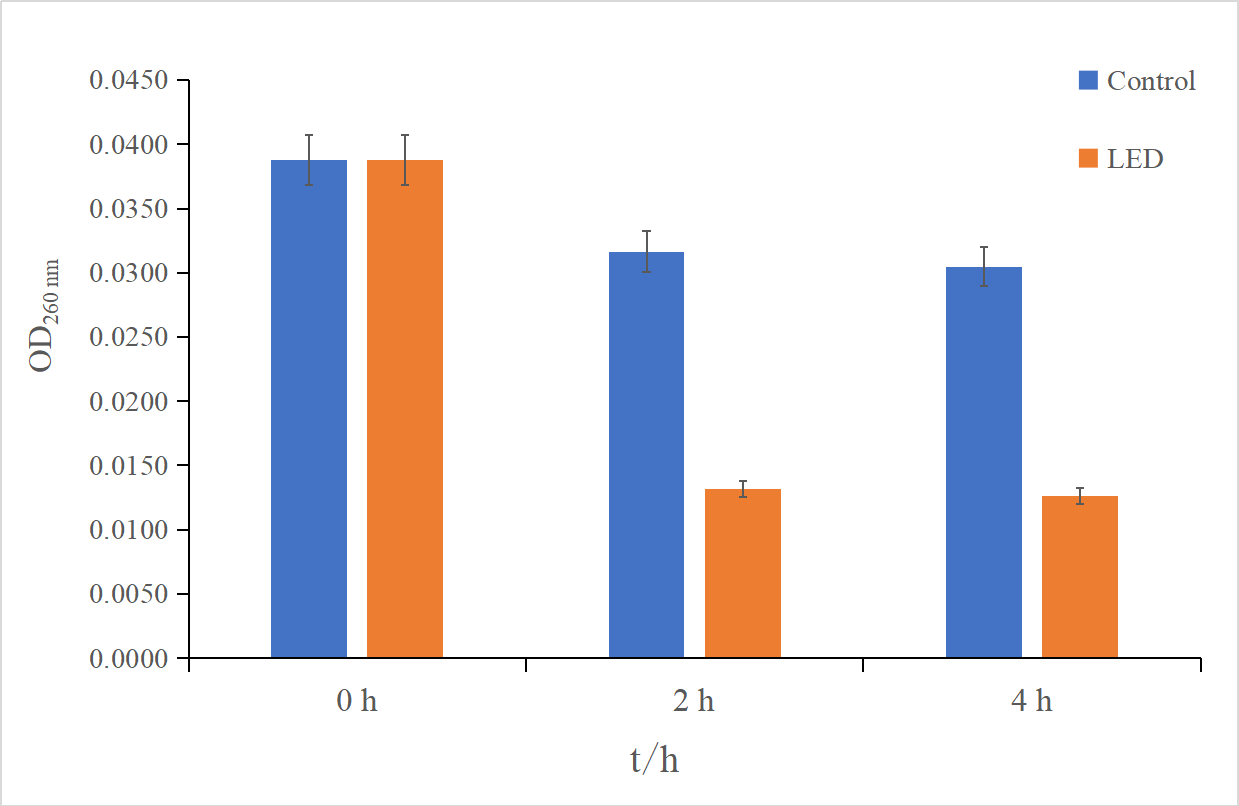

Supplement: Supplementary file 1 [file Image_1.TIF]
